# Therapeutic effect of intensive granulocyte and monocyte adsorption apheresis combined with thiopurines for steroid- and biologics-naïve Japanese patients with early-diagnosed Crohn’s disease

**DOI:** 10.1186/1471-230X-14-124

**Published:** 2014-07-11

**Authors:** Takumi Fukuchi, Hiroshi Nakase, Satoshi Ubukata, Minoru Matsuura, Takuya Yoshino, Takahiko Toyonaga, Keiji Shimazu, Hideaki Koga, Hiroshi Yamashita, Dai Ito, Kiyoshi Ashida

**Affiliations:** 1 Department of Gastroenterology and Hepatology, Osakafu Saiseikai Nakatsu Hospital, 2-10-39 Shibata, Kita-ku, Osaka 530-0012, Japan; 2 Department of Gastroenterology and Hepatology, Graduate School of Medicine, Kyoto University Hospital, 54 Kawahara-cho, Shogoin, Sakyo-ku, Kyoto 606-8507, Japan; 3 Department of Nephrology, Osakafu Saiseikai Nakatsu Hospital, 2-10-39 Shibata, Kita-ku, Osaka 530-0012, Japan

**Keywords:** Crohn’s disease, Granulocyte and monocyte adsorptive apheresis, Thiopurines, Mucosal healing

## Abstract

**Background:**

Early induction with biologics can reduce complications in patients with Crohn’s disease (CD) and improve their quality of life. The safety of biologics, however, is uncertain. Granulocyte and monocyte adsorptive apheresis (GMAA) is a natural biologic therapy that selectively removes granulocytes and monocytes/macrophages and has few severe adverse effects. The effects of GMAA on patients with early-diagnosed CD are unclear. We investigated the effects of GMAA combined with thiopurines on patients with early-diagnosed CD.

**Methods:**

Twenty-two corticosteroid- and biologic-naïve patients with active early-diagnosed CD were treated with intensive GMAA (twice per week) combined with thiopurines administration. Active early-diagnosed CD was defined as follows: (i) within 2years after diagnosis of CD, (ii) with no history of both surgical treatment and endoscopic dilation therapy, and (iii) Crohn’s Disease Activity Index (CDAI) was higher than 200. We investigated the ratios of clinical remission defined as CDAI was less than or equal to 150 at 2, 4, 6 and 52weeks and mucosal healing defined as a Simplified Endoscopic Activity Score for Crohn’s Disease (SES-CD) as 0 at 6 and 52weeks. Adverse events were recorded at each visit.

**Results:**

The ratios of clinical remission at 2, 4, and 6 weeks were 6 of 22 (27.2%), 12 of 22 (54.5%), and 17 of 22 (77.2%), respectively. At 52 weeks, 18 of 21 patients (81.8%) were in clinical remission. The ratios of mucosal healing at 6 and 52 weeks were 5 of 22 (22.7%) and 11 of 22 (50%), respectively. The difference in the mucosal healing ratio was significant between 6 and 52 weeks (p = 0.044). No serious adverse effects were observed during this study.

**Conclusions:**

Combination therapy with intensive GMAA and thiopurines administration rapidly induced high remission in patients with active early-diagnosed CD without serious adverse effect. Mucosal healing was observed in 50.0% of enrolled patients. This combination therapy might be a rational option for patients with early-diagnosed CD.

## Background

Crohn’s disease (CD) is a chronic inflammatory disorder of unknown etiology. The current practice guidelines are designed to maintain remission with optimal medication after induction therapy due to the potential for repeated relapse in patients with CD [1,2].

Corticosteroids effectively suppress acute CD flares. Approximately 60% of patients will have a complete response during the short-term outcome (30 days) of a first course of steroids [3]. Long-term exposure to corticosteroids, however, is problematic due to dependency and/or the development of resistance [4].

In the last 15 years, the advent of biologic therapies, such as anti-tumor necrosis factor (TNF)-α, has improved the management of refractory CD. In addition, the efficacy of TNF-α antagonists suggests a new concept that early induction with intensive therapy may reduce complications associated with conventional treatment and improve the patient’s quality of life [5]. In this regard, intensive therapy with early use of this biologic agent has been proposed and is known as ‘top-down’ therapy. Early use of anti-TNF therapy, however, is associated with the development of serious life-threatening infections in addition to other well-documented hematologic, immunologic, cardiovascular, and malignant adverse effects [6]. Therefore, whether long-term use of anti-TNF therapy is suitable for all patients with CD remains unclear.

A 2-year open-label randomized trial by D’Haens et al. [5] showed that short-term infliximab (IFX) combined with azathioprine (AZA) or 6-mercaptopurine was more effective than conventional management for the induction of remission and reduction of corticosteroid use in patients recently diagnosed with CD, and patients treated with early combined immunosuppression had consistently higher rates of remission than controls within the first year of treatment. The findings from this study suggest that early use of immunomodulators following optimal induction therapies has favorable effects on active CD. In addition, recent Hungarian population-based inception cohort [7] showed that a reduction in the surgical ratio in patients with CD was independently associated with increased early use of thiopurines. Thus, the development of promising induction therapies with thiopurines is expected for patients with CD to avoid complications related to various drugs as much as possible.

The therapeutic effects and safety of granulocyte and monocyte adsorptive apheresis (GMAA) with the Adacolumn (JIMRO, Takasaki, Gunma, Japan) for patients with inflammatory bowel disease (IBD) have been much focused because this system can be expected to be a natural biologic therapy in that selectively removing granulocytes and monocytes/macrophages from the peripheral blood leads to decreased production of inflammatory cytokines [8-10]. Recent data demonstrated that ulcerative colitis (UC) patients treated with a twice-a-week regimen (intensive GMAA) could achieve significantly higher ratios of clinical remission and mucosal healing compared with an once-a-week regimen (weekly GMAA) [11,12]. In addition, it was reported that therapeutic effect of GMAA with 10 sessions on active UC patients was superior to that with 5 sessions [13,14]. Thus, intensive GMAA with 10 sessions might be an optimal therapy for the induction of remission in patients with CD as well as UC.

In the present study, we prospectively followed patients with newly diagnosed CD for 1year after administering intensive GMAA combined with thiopurines, focusing on clinical remission and endoscopic activity.

## Methods

### Patients

From January 2010 to August 2012, a total of 22 corticosteroid- and biologics-naïve patients with moderate to severe active early-diagnosed CD were enrolled in the study. Early-diagnosed CD was defined as follows: (i) diagnosis within the past 2years, (ii) patients with CD and a past history of surgery and endoscopic dilation for CD were excluded. The diagnosis of CD was based on clinical, endoscopic, radiologic, and histologic findings. Fecal bacteria culture yielded no specific pathogens in any of the patients. Patients older than 17 years of age with active early-diagnosed CD were consecutively recruited if they had newly or relapsing active disease. Moderate-to-severe active CD was defined as a Crohn’s Disease Activity Index (CDAI) [15] higher than 200. Oral aminosalicylates were only permitted if given at a stable dose. None of the patients had undergone previous treatment with corticosteroids or TNF-α antagonists (Table 1).

**Table 1:** Clinical parameters of patients with early-diagnosed Crohn’s disease treated by intensive granulocyte and monocyte adsorptive apheresis combined with thiopurines therapy

**Age (years)**	39.5 ± 2.9
**Sex (men/women)**	18/4
**Disease duration (months)**	5.8 ± 1.8
**Disease location**	
** Colon**	13 (59.1)
** Colon/Ileum**	3 (13.6)
** Ileum**	6 (27.3)
**Disease behavior**	
** Inflammatory**	22 (100)
** Structure**	1 (4.5)
** Perforation**	0
**Perianal disease**	2 (9.1)
**Previous treatment**	
** 5-ASA**	5 (22.7)
** Thiopurines**	0
** Corticosteroids**	0
** Biologics**	0
**CDAI**	260.0 ± 10.5
**SES-CD**	9.4 ± 0.9
**CRP (mg/dl)**	3.5 ± 0.7

Number of patients is shown for sex, disease location, disease behavior, perianal disease and previous treatment. Data are presented as mean ± SE for age, disease duration, CDAI, SES-CD, and CRP. 5-ASA 5-aminosalicylate; CDAI, Crohn’s disease activity index; SES-CD, Simple endoscopic score of Crohn’s disease. Values in parenthesis on vertical line are percentages of all 22 patients with early-diagnosed CD.

### Treatment

All 22 patients with early-diagnosed CD were treated by GMAA combined with thiopurine administration. Intensive GMAA treatment (twice per week, 10 sessions) was performed as previously described [11,12,16] for all patients with CD. Blood was accessed via the antecubital vein in one arm, and from the outflow blood was returned to patients via the antecubital vein in the other arm through 19 gauge needles. The apheresis session was performed at a flow rate 30 mL/min for 60 min, aiming to expose 1800 mL blood/session. Anti-coagulation for GMAA therapy was induced by heparin sodium. Thiopurines were administered to all 22 patients at the start of GMAA therapy. The dose of thiopurines was adjusted to aim for a white blood cell count < 5000/ml. Patients responding to treatment with intensive GMAA kept receiving thiopurines drugs for 52 weeks from the end of the intensive GMAA. Mean daily dose of thiopurines was 30.0 ± 2.5 mg. During this study, adverse events were recorded at each visit.

The protocol and patients’ informed consent forms were reviewed and approved by the Institutional Review Board at Osakafu Saiseikai Nakatsu Hospital. The study was conducted in accordance with the Declaration of Helsinki, the consolidated Good Clinical Practice Guidelines, and the applicable regulatory requirements.

### Assessment of endoscopic severity

All 22 patients with early-diagnosed CD underwent ileocolonoscopic examinations before, and at 6 and 52 weeks after treatment. Endoscopic severity of CD was assessed using the Simplified Endoscopic Activity Score for Crohn’s Disease (SES-CD) [17].

### Assessment

Primary efficacy was evaluated based on the clinical remission rate at 2, 4, and 6weeks during intensive GMAA, and 13, 26, and 52weeks after starting treatment. Clinical remission was defined as CDAI ≤ 150. Secondary efficacy was evaluated based on SES-CD, complete mucosal healing rate, and mean C-reactive protein (CRP) values at weeks 6 and 52. Complete mucosal healing was defined as a SES-CD of 0 [18]. If patients received rescue treatment such as intensive GMAA again, corticosteroids, or biologics because of clinical relapse of CD, their treatment was considered to have failed.

### Statistical analysis

Data are presented as mean ± SE. Categorical and continuous data were compared using a two-tailed Fisher exact test and Student’s *t*-test. A p value of less than 0.05 was considered statistically significant.

## Results

### Clinical characteristics

The clinical characteristics of patients with early-diagnosed CD enrolled in this study are summarized in Table 1. Mean patient age was 39.5 ± 2.9 years, mean patient disease duration was 5.8 ± 1.8 months (range 1–32 months), and mean CDAI was 260.0 ± 10.5. Disease location was the colon in 13 (59.1%), colon/ileum in 3 (13.6%), and ileum in 6 (27.3%). Mean SES-CD was 9.4 ± 0.9 (range 5–19). Of the 22 patients, 5 patients (22.7%) were treated with a stable dose (3000 mg) of 5-ASA. None of patients enrolled in the study had been treated previously with corticosteroids, thiopurines, or biologics (Table 1).

### Clinical efficacy

**Figure 1:**
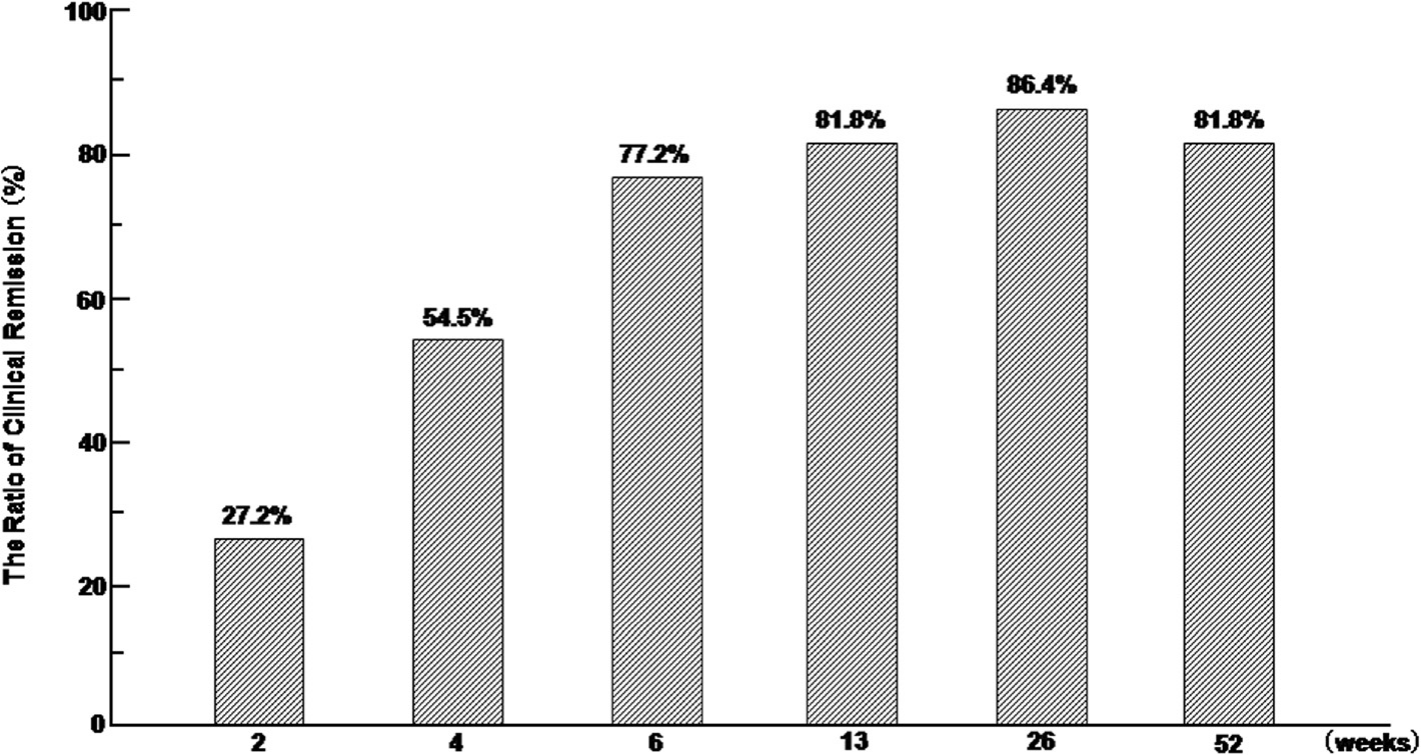
**Maintenance of clinical remission and response in patients responding to combination therapy with intensive GMAA and thiopurines.** Bar graph showing the percentage of patients in remission (CDAI < 150) over time.

The ratios of clinical remission at 2, 4, and 6weeks were 6 of 22 (27.2%), 12 of 22 (54.5%), and 17 of 22 (77.2%), respectively (Figure 1). Mean time and number of GMAA sessions to clinical remission were 28.0 ± 2.9 days and 7.3 ± 0.6 sessions, respectively, in patients with CD who achieved clinical remission during intensive GMAA. One patient with colonic structure and 1 of 2 patients with perianal disease did not achieve clinical remission during intensive GMAA. The ratios of clinical remission at 13weeks and 26weeks were 18 of 22 (81.8%) and 19 of 22 patients (86.4%), respectively. At 52 weeks, 18 of 22 patients (81.8%) were in clinical remission (Figure 1).

During this study, two patients were treated with adalimumab because of non-response to intensive GMAA and two patients required intensive GMAA again because of CD flares.

### Evaluation of mucosal healing

**Figure 2:**
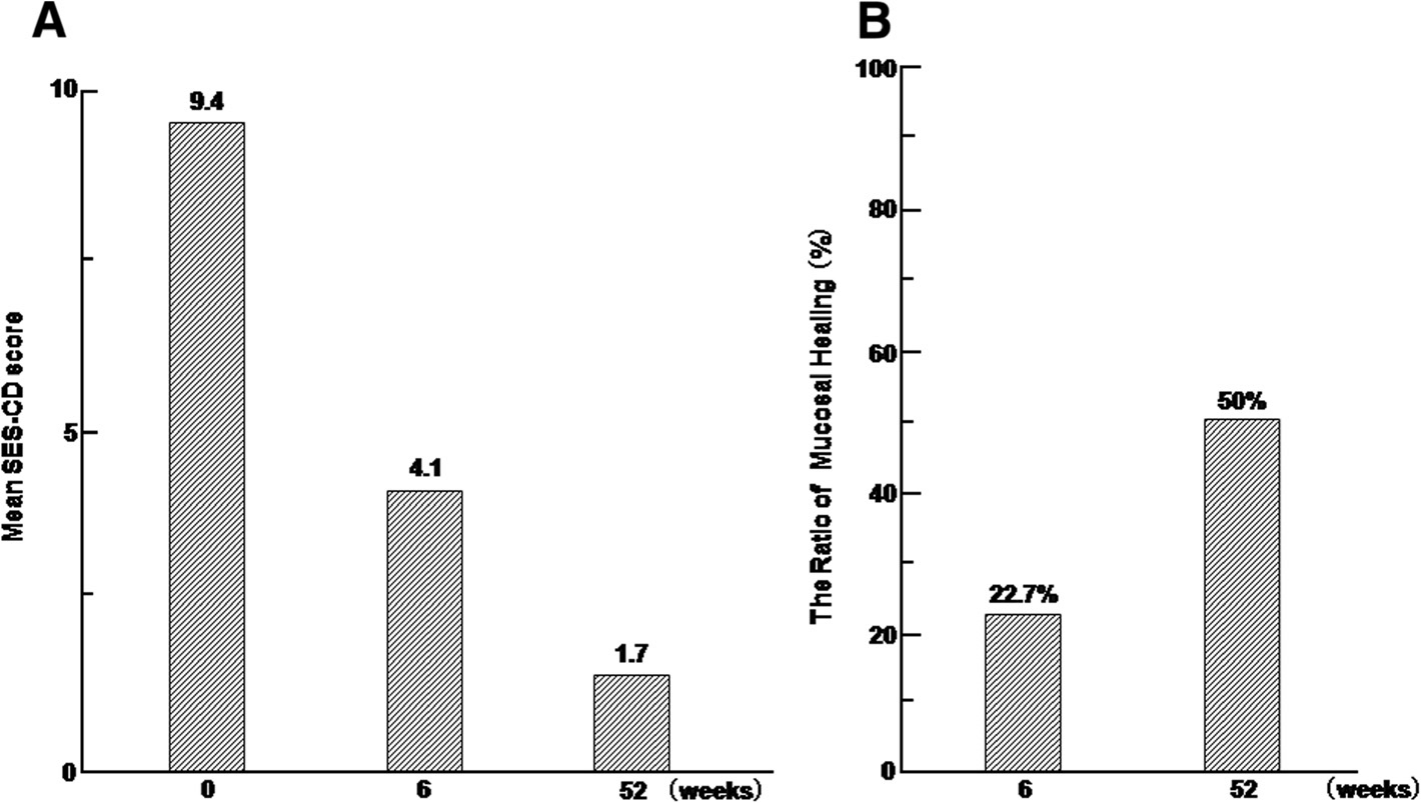
**The change of endoscopic findings in the patients with early-diagnosed Crohn’s disease by combination therapy with intensive GMAA and thiopurines. (A)** Bar graph illustrating the mean Activity Score for Crohn’s Disease (SES-CD) scores and **(B)** the ratio of mucosal healing at 6 and 52 weeks after starting treatment.

Mean SES-CD scores in all patients are shown in Figure 2-A. Mean SES-CD scores were compared at three time points (0, 6, and 52weeks). The scores were significantly different between 0 and 52weeks. In addition, the ratios of mucosal healing at 6 and 52weeks were 5 of 22 (22.7%) and 11 of 22 (50%), respectively (Figure 2-B). The ratio of mucosal healing between 6 and 52weeks were significantly different.

### Change in CRP values by treatment

At 6weeks after starting treatment, the mean CRP for all patients decreased significantly from 3.5 ± 0.7 to 0.4 ± 0.1mg/dl (p = 0.00003). At 52weeks, mean CRP in patients followed for 52weeks was 0.2 ± 0.1 mg/dl. Serum CRP levels differed significantly between before treatment and 52weeks (p = 0.0002).

### Safety

Two patients complained of nausea, but their symptoms improved following a dose reduction of thiopurines. No other serious adverse effects were observed during this study.

## Discussion

In the present study we firstly demonstrated that intensive GMAA in combination with thiopurines resulted in higher ratios of clinical remission and mucosal healing at 52weeks in corticosteroid- and biologics-naïve patients with early-diagnosed CD. This clinical study was of small size, however, expected to demonstrate the efficacy and safety of the combination therapy of GMAA and thiopurines.

The Study of Biologic and Immunomodulator Naïve Patients in Crohn’s Disease (SONIC) demonstrated that IFX in combination with AZA was superior to IFX monotherapy for patients not treated previously with AZA [19]. An open-labeled clinical trial reported by D’Haens et al. [5] showed the combination therapy (immunomodulators in combination with three infusions of IFX at 0, 2, and 6 weeks) resulted in a significantly higher clinical remission rate in patients with early-diagnosed CD compared with conventional medication. These data suggest the importance of optimal induction therapies with thiopurines for patients with early-diagnosed CD.

In this study, we performed intensive GMAA (twice per week) as an alternative to weekly GMAA as an induction therapy. Previous data on the ratios of the induction of remission in active CD refractory to corticosteroids by weekly GMAA vary (30%-60%) [20-22]. Thus, the efficacy of GMAA therapy on active CD patients as an induction therapy has not been established. On the contrary, the remarkable usefulness of intensive GMAA for the induction of remission and mucosal healing in patients with active UC compared with weekly GMAA suggests that the therapeutic effects of GMAA are associated with frequency of therapy per week [11,12]. In fact, our data showed that this combination therapy as an induction treatment in patients with active CD showed a remission rate comparable to that of corticosteroids or biologics reported in controlled trials [3,5,19]. The therapeutic outcome of intensive GMAA in this study might be due to not only the GMAA frequency per week, but also the population of eligible patients (corticosteroid- and biologics-naïve early-diagnosed CD). In this regard, intensive GMAA with thiopurines can be a promising induction therapy for patients with early-diagnosed CD. Additionally, there were no adverse effects related to GMAA in this study. The induction of remission by GMAA in the patients with early-diagnosed CD would have the advantage in the point of avoiding adverse effects related to corticosteroid and biologics.

Several studies have demonstrated the efficacy of immunomodulators to prevent relapse in patients with CD and showed that early use of immunomodulators decreases the cumulative surgical rate in patients with CD [5,7,23]. In the present study, maintenance treatment with thiopurines increased the clinical remission rate in patients with early-diagnosed CD after treatment with intensive GMAA. Also, our data demonstrated an extremely high clinical remission rate at 52 weeks (81.8%) in CD patients treated with combination therapy in comparison with previous reports. In this regard, the difference in the number and characteristics of the enrolled patients may account for the differences in the clinical remission ratio between the present study and previous data. In this study, how treatment with thiopurines could involve in having CD patients keep remission remains unclear. Therefore, future clinical trial comparing the relapse-free survival of CD patients achieving remission by GMAA who receive thopurines with those who do not, should be required. This head to head trial might elucidate who requires thiopurines among patients with early-diagnosed CD achieved clinical remission after GMAA.

It should be noted that recent two clinical trials demonstrated that very early administration of AZA is not effective for corticosteroid free-clinical remission in patients with newly diagnosed CD [24,25]. However, one trial (the GETAID group) showed that approximately 60% of CD patients in the conventional management group finally required any immunosuppressive drugs for corticosteroid free-clinical remission in a 3-year study period and the occurrence of perianal lesions was significantly lower in the AZA-treated group than in the conventionally-treated group [24]. In addition, another trial (the AZTEC study group) reported that early AZA therapy is more effective for preventing a moderate to severe relapse in a post hoc analysis [25]. Thus, these two reports did not necessarily deny the effect of AZA in the treatment for CD patients [24,25], but we should realize that these two clinical trials suggest the importance of identifying when AZA would be started in the population of CD patients whose disease activity becomes progressive without optimal immunosuppressive treatments.

Recently, mucosal healing has been focused as a predictive marker of sustained remission irrespective of no demonstration of clinical benefit of achieving complete mucosal healing [18,26,27]. Rutgeerts et al. [26] reported the value of an endoscopic score to predict recurrent CD. In addition, Baert et al. [18] demonstrated the importance of mucosal healing defined as SES-CD 0, based on a significant difference in the steroid-free remission ratio after therapy between patients with CD with SES-CD 0 and SES-CD 1–9. In the present study, we prospectively examined mucosal healing in patients with CD treated with combination therapy. We observed that ratios of mucosal healing at 6 and 52 weeks were 22.7% and 50.0%, respectively, and that at 52 weeks was comparable to data reported by D’Haens et al. [27] and Colombel et al. [19]. Thus, our and D’Haens et al. [27] data might suggest the usefulness of early induction with thiopurines in biologics-naive patients with CD after induction with optimal therapy, such as intensive GMAA, although we should consider that the timing of the evaluation of mucosal healing varied depending on the medical treatment and the characteristics of the enrolled patients.

The overall incidence of adverse events was related to thiopurines, and not intensive GMAA. It was reported that the combination of thiopurines and anti-TNF biologic agents increases the relative risk of serious and opportunistic infections [28]. Also, the use of corticosteroids may further increase the relative risk of infections [3]. GMAA is a natural biologic therapy for the patients with IBD and has few severe adverse effects. In the point of avoiding serious side effect and being performed repeatedly and safely, GMAA might be more advantageous than anti-TNF or corticosteroids. Therefore, intensive GMAA is considered to be an induction therapy alternative to corticosteroids and three infusions of IFX (at 0, 2, and 6 weeks).

## Conclusions

Our findings showed that the combination therapy with intensive GMAA and thiopurines was an effective and well-tolerated therapeutic option for inducing and maintaining clinical remission in patients with early-diagnosed CD. The data, however, were derived from an open study including only a small number of patients, and did not address whether intensive GMAA with thiopurines for biologics-naïve patients with early-diagnosed CD was superior to corticosteroids and IFX. Therefore, future prospective studies with larger numbers of patients are required to assess the efficacy and safety of this combination therapy.

## Abbreviations

CD: Crohn’s disease; TNF: Tumor necrosis factor; IFX: Infliximab; AZA: Azathioprine; GMAA: Granulocyte and monocyte adsorptive apheresis; IBD: Inflammatory bowel disease; UC: Ulcerative colitis; CDAI: Crohn’s disease activity index; SES-CD: Simplified endoscopic activity score for Crohn’s disease; CRP: C-reactive protein.

## Competing interests

The authors declare that they have no competing interests.

## Authors’ contributions

TF, SU: Patients management, conception, study design, acquisition and interpretation of the data, statistics, drafting, and preparation of the final. HN, MM, TY, and TT: Conception, study design, drafting, and review of the final manuscript. KS, HK, HY, DI and KA: Patients management, acquisition and interpretation of the data, and review of the final manuscript. All authors read and approved the final manuscript.

## Pre-publication history

The pre-publication history for this paper can be accessed here:

http://www.biomedcentral.com/1471-230X/14/124/prepub
